# Guidelines to PET measurements of the target occupancy in the brain for drug development

**DOI:** 10.1007/s00259-016-3476-4

**Published:** 2016-08-11

**Authors:** Akihiro Takano, Andrea Varrone, Balázs Gulyás, Piero Salvadori, Antony Gee, Albert Windhorst, Johnny Vercouillie, Guy Bormans, Adriaan A. Lammertsma, Christer Halldin

**Affiliations:** 1 Department of Clinical Neuroscience, Centre for Psychiatric Research, Karolinska Institutet, Stockholm, Sweden; 2 CNR Istituto di Fisiologia Clinica, Pisa, Italy; 3 Department of Chemistry and Biology, Division of Imaging Sciences and Biomedical Engineering, Kings College London, London, UK; 4 Department of Radiology & Nuclear Medicine, VU University Medical Center, Amsterdam, The Netherlands; 5 Université François Rabelais de Tours, UMR Inserm U930, Tours, France; 6 Nuclear Medicine and Molecular Imaging, Department of Imaging and Pathology, KU Leuven, Leuven, Belgium

**Keywords:** Drug development, Target occupancy, Positron emission tomography

## Abstract

This guideline summarizes the current view of the European Association of Nuclear Medicine Drug Development Committee. The purpose of this guideline is to guarantee a high standard of PET studies that are aimed at measuring target occupancy in the brain within the framework of development programs of drugs that act within the central nervous system (CNS drugs). This guideline is intended to present information specifically adapted to European practice. The information provided should be applied within the context of local conditions and regulations.

## Background

Positron emission tomography (PET) is a molecular imaging tool well suited for in vivo imaging and quantifying receptors, transporters, and enzymes in the brain that can be potential targets for CNS drugs. Within drug development programs at the industrial level, the average cost for a drug to be brought to the market is approximately US$ 1 billion, and the time needed for the process is typically up to 15 years [1]. In addition, based on estimates of the US Food and Drug Administration (FDA), there is less than a 10 % chance for a drug entering clinical trials to reach the market, often because of lack of efficacy, undesirable side-effects, or inappropriate kinetics [2]. In the CNS, despite the presence of several hundreds of potential targets for new drugs, the functional relevance of most of the proteins in the human brain is not fully understood, especially in relation to the doses of new drugs. Inappropriate selection of doses, namely too high or too low doses, may lead to failure of the drug development in phases 2 and/or 3. Measurement of target occupancy in the brain in vivo is expected to be useful for an appropriate dose setting.

These guidelines are primarily focused on the use of positron emission tomography as a molecular imaging tool to investigate the fraction of the total target population (receptor, transporter or enzyme, etc.) in the brain that is occupied by a CNS drug [3].

In these guidelines, we assume that standard stand-alone PET systems and appropriate PET reconstruction algorithms are implemented and used for the PET occupancy study. Regular calibration of the PET systems should be performed to validate the data acquisition. When PET/CT or PET/MRI is considered for use, extra cautions for each modality’s characteristics such as radiation from CT and issues of the attenuation and scatter corrections related to MRI should be taken into consideration. The bias introduced by different PET reconstruction algorithms should be also considered before the study starts. The issues related to validation of the modalities should be checked elsewhere.

We hope that these guidelines would help all researchers who are interested in the target occupancy study of CNS drugs, especially to researchers who aim to establish and/or improve the collaborative PET studies for drug development between academic sites and pharmaceutical companies.

All preclinical and clinical PET occupancy studies should have ethical approvals from relevant committees before the studies start. Clinical studies should follow the declaration of Helsinki and possibly organize the central recordings of the PET studies.

## Key issues to consider for the PET occupancy study

### Selection of appropriate radioligands

The following three criteria are recommended for radioligands used in PET occupancy studies.

1. High specific binding and selectivity for the target
  If the ratio of specific to nonspecific binding is low, the outcome measures will be less robust. If a radioligand binds to several targets (i.e., has low selectivity), the occupancy calculated for the test drug may be underestimated, if the latter is selective for a single target. If test drug and radioligand both bind competitively to off-target sites, the occupancy might be calculated incorrectly. If possible, information on in vitro affinities for both target and off-target sites should be obtained prior to PET occupancy studies.
2. Well-established quantitative methods for measuring radioligand binding
  Quantitative methods for measuring radioligand binding should be established well before PET occupancy studies. To calculate occupancy, outcome measures of these methods should preferably reflect specific binding to the target (e.g., binding potential (*BP*_ND_) or specific distribution volume (*V*_S_)) [4]. Reversibility and level of non-displaceable binding of the tracer, together with total length and framing of the dynamic PET measurement should be established in advance. Radioligands for which *BP*_ND_ or *V*_S_ are difficult to estimate are discussed later in section E.
3. Low test-retest variability of outcome measures of the radioligand
  Although there is no definite threshold for the test-retest variability needed for occupancy studies, clearly variability should be as low as possible. When test-retest variability is high, more data points are needed for robust characterization of the dose-occupancy curve. Apart from the radioligand itself, test-retest variability depends on several other factors such as the PET facilities and auxiliary equipment used. In any case, test-retest variability should be measured prior to or as part of the occupancy study.

### Selection of subjects

#### Experimental animals (nonhuman primates, pigs, rats, etc.)

If experimental animals are available at the local PET facility, preclinical PET occupancy studies can be considered before studies in human subjects. Preclinical PET studies should only be performed in a complimentary and/or bridging manner to the clinical PET occupancy study, i.e., when full occupancy cannot be achieved in humans because of safety reasons. It should be considered that there may be species differences in the kinetic behaviour and metabolism of the radioligand and the test drug. Therefore, preclinical studies should be designed in such a way that they are relevant for the human situation. The choice of experimental animals is essential in ensuring that results of occupancy studies can be translated to human subjects. Although the accessibility to the nonhuman primates (NHP) is limited in some parts of Europe, non-human primates are typically considered as a suitable model to predict the behaviour of a drug and a PET radioligand in the human brain.

#### Human subjects (healthy volunteers, patients), gender and age

In general, healthy volunteers are appropriate for human PET occupancy studies, at least in the initial stages of developing a new drug. If the target density is expected to be variable during the menstrual cycle or when other gender factors could play a role, inclusion/exclusion criteria or the schedule of PET measurements should be adjusted accordingly. Inclusion/exclusion criteria of healthy volunteers should be defined for each PET occupancy study, considering possible confounding variables for each target.

Certain drug targets in the brain, e.g., dopamine receptors are known to decrease with age. Nevertheless, as it can be considered to be stable during the short time interval of a PET occupancy study, i.e., within 1–2 months, age effects on occupancy measurements usually are negligible (for repeat measurements in the same healthy subjects).

Clearly, if a test drug is intended for a certain age range and/or gender, e.g., test drugs for dementia, and pharmacokinetic parameters are expected to depend on age and/or gender, occupancy studies should be performed in the appropriate target population. In addition, when patients are included in an occupancy study, it is always important to verify whether previous chronic drug treatment has had any effects on the binding of the radioligand to the target (e.g., because of very slow dissociation from the target or by altering expression of the target).

### Designing and scheduling of the study

#### Preclinical PET occupancy study

**Fig. 1:**
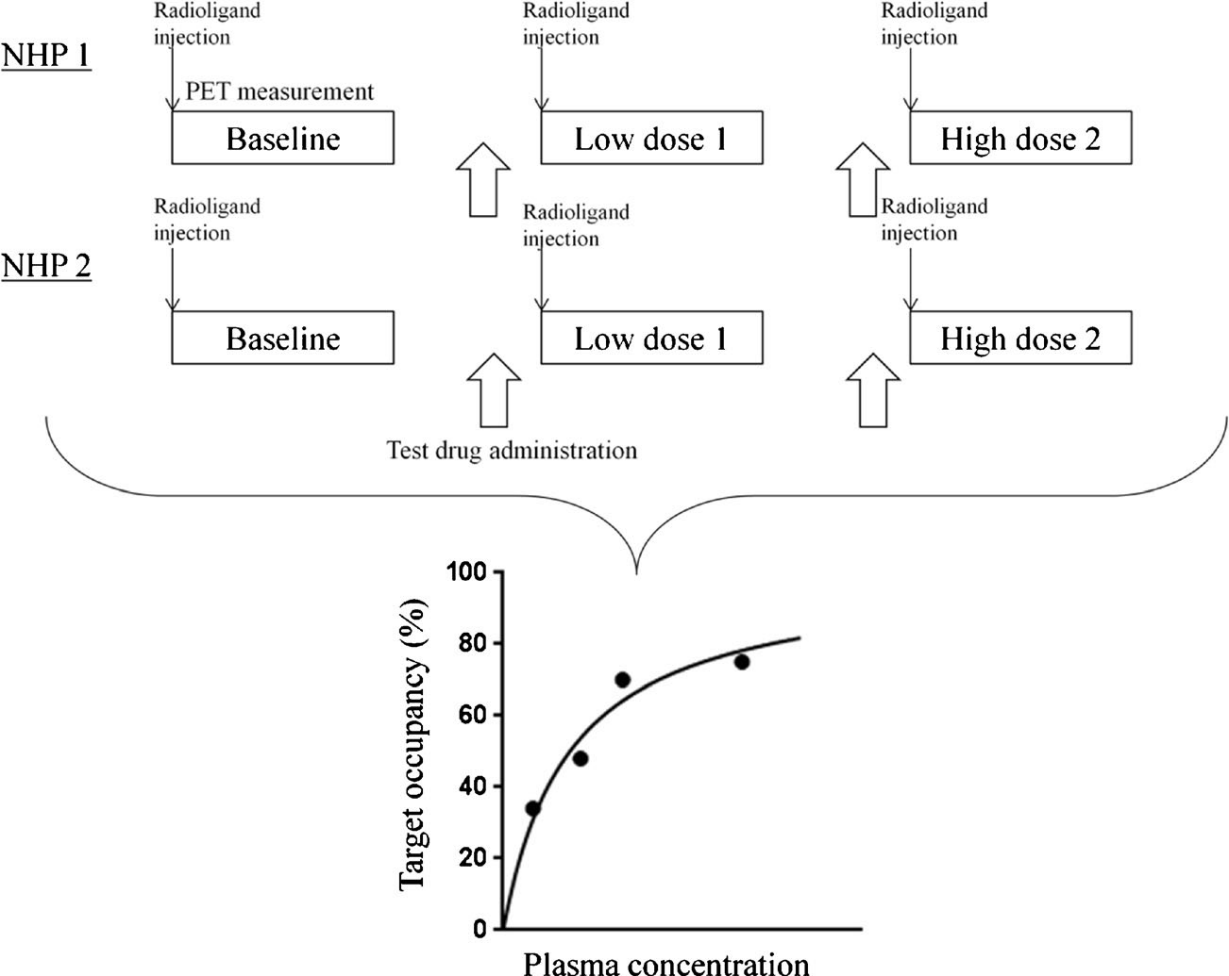
Example of a schedule of a PET occupancy protocol using a C-11 labelled ligand. Two nonhuman primates are enrolled in the study. A total of three PET measurements, one baseline measurement followed by two pretreatment (one at low and one at higher dose) measurements, are performed in each nonhuman primate on the same day. In the second nonhuman primate, the drug dose can be adjusted based on the results of the first (adaptive design). According to this schedule, four occupancy data points can be obtained in 2 days. If the radioligand is labelled with F-18, only one PET measurement can be performed per day due to the longer half-life and more days are required to obtain the same four occupancy data points

At least one baseline PET measurement (i.e., when no test drug is administered before PET measurement) and one pretreatment PET measurement (i.e., the test drug is administered before PET measurement) should be performed for each experimental animal. During PET measurements, vital signs, such as blood pressure, heart rate, ECG, and blood oxygenation should be monitored to evaluate potential side effects. In principle, even when a preclinical PET occupancy study is performed in the early phase of drug development, ideally safety data of the test drug should be available prior to the PET study, especially in the case of NHP. Although a wide range of doses for the test drug are investigated in a PET occupancy study, the highest dose should be lower than the maximal safe dose. An example PET schedule using NHP, in which four occupancy data points are obtained in 2 days, is shown in Fig. 1.

The study design and scheduling of the preclinical PET occupancy study depends on the following factors:

- Radioligand synthesis at the facility
  How many times per day can the radioligand be synthesized?
  How many times per day can the radioligand be synthesized?
- Availability of experimental animals
  What is the maximum allowable time per experiment?
  What is the maximum allowable number of measurements per experimental animal?
  Are studies without anaesthesia possible?
  Type of anaesthesia, its interaction with radioligand and/or test drug. Preferably in vitro data about the effects on the radioligand binding by anesthesia should be available prior to PET occupancy study.
- Characteristics of the radioligands
  The half-life of the radionuclide (e.g., C-11 or F-18) used for labelling, defining the minimum interval between measurements (at least 5 half-lives).
  Brain kinetics of the radioligand, defining minimum duration of a PET measurement, e.g., 60 min for [^11^C]raclopride (dopamine D_2_ receptor), and requiring a radionuclide with a suitable half-life.
- Blood sampling and metabolite analysis
  For many PET radioligands (i.e., all radioligands without a valid reference region), measurement of the arterial input function is necessary for quantitative analysis of occupancy studies, raising the following issues:
    Is arterial sampling feasible?
    What is the maximum allowable blood volume that can be withdrawn (e.g., in repeat measurements)?
    Is equipment for metabolite analysis and measurement of protein binding available?
- Pharmacokinetics of the test drug
  Routes of drug administration, i.e., orally, intravenous or intramuscular?
  In case of intravenous administration, bolus, bolus plus constant infusion, slow infusion, or acute vs chronic dosing?
  Time to reach maximal plasma level of the drug (T_max_) and time at the trough level of the drug (T_trough_).
  Presence of active metabolites?
  Timing of the pre-treatment PET measurement(s): aimed for T_max_ and/or T_trough?_
  Multiple pre-treatment PET measurements to investigate the time course of the target occupancy?
  Effects of the test drug on cerebral blood flow. PET measurements should be performed at the physiological steady state, i.e., not when cerebral blood flow undergoes (rapid) changes due to the test drug.

#### Clinical PET occupancy study

Safety data of the test drug should be available prior to the PET study. At least one baseline PET measurement (i.e., when no test drug is administered before PET measurement) and one pretreatment PET measurement (i.e., the test drug is administered before PET measurement) should be performed per subject. Theoretically, the relationship between target occupancy and dose/plasma concentration of the drug can be established from just a single occupancy data point according to the law of mass action, provided the drug only interacts with a single binding site. However, considering the presence of noise in the PET data, it is advisable to have as many points as possible to examine the relationship between dose or plasma concentration of the drug and target occupancy. Ideally, several different doses should be used in order to cover the entire range from low to high occupancy levels. In addition, to account for intersubject variability, it is recommended to scan several subjects with the same dose.

The study design of a clinical PET occupancy study depends on the following factors:

- Radioligand synthesis at the facility.
  In case of C-11, how many times per day can the radioligand be synthesized?

b. Radiation dosimetry
  The total number of PET measurements is limited by the radiation absorbed dose received by the subjects. Generic dosimetry tables are available for both ^18^F and ^11^C labelled brain tracers, for adults indicating effective doses of 4.3 (^11^C) and 28 (^18^F) μSv/MBq, respectively [5], illustrating that more repeat studies can be performed using an ^11^C labelled tracer. For novel ligands, as it depends on the kinetics of the radioligands [6, 7], the absorbed dose of individual radioligand should be investigated. The ICRP has published absorbed dose constraints for medical exposure for volunteers in biomedical research, depending on its benefit to society ranging from 0.1 mSv for minor benefit to 10 mSv for moderate benefit, and > 10 mSv only for substantial benefit [8]. Research studies in healthy volunteers can be considered of intermediate to moderate benefit, depending on the type of the study. For each clinical PET occupancy study, the total number of PET measurements per subject together with injected radioactivity per PET measurement should result in a radiation absorbed dose, which is accepted by the local radiation safety committee. When PET/CT is used, additional radiation exposure from CT should be added for the radiation absorbed dose. PET/MRI or digital PET might help to lead to less radiation exposure in the future.

c. Availability of human subjects
  Age, gender, healthy volunteers or patients; in general, dose-occupancy studies are first performed in healthy subjects, but the findings need to be substantiated in a target patient population.
  In those cases in which it is difficult to perform a baseline PET measurement, e.g., in cases of drug naïve patients with schizophrenia, a normal database can be used as a reasonable alternative for the baseline measurements. However, this approach should be considered with caution considering the potential differences between the two groups of subjects.

d. Characteristics of the radionuclide
  The half-life of the radionuclide (e.g., C-11 or F-18) used for labelling, defining the minimum interval between measurements (at least 5 half-lives) and the maximum number of PET measurements per subject.
  Brain kinetics of the radioligand, defining the minimum duration of a PET measurement, e.g., 60 min for [^11^C]raclopride (dopamine D_2_ receptor), which requires a radionuclide with a suitable half-life.

e. Blood sampling and metabolite analysis
  For many PET radioligands (i.e., all radioligands without a valid reference region), arterial input is essential for quantitative analysis of occupancy levels.
    Is arterial sampling feasible?
    Is the amount of blood to be withdrawn acceptable?
    Is equipment for metabolite analysis and measurement of protein binding available?

f. Pharmacokinetics of the test drug
  Safety data, single dose or multiple doses.
  Time to reach the maximum plasma level of the drug.
  Presence of active metabolites
  Timing of pre-treatment PET measurement(s): aimed for T max and/or T trough? Multiple pretreatment PET measurements to investigate the time course of target occupancy.
  Effects of the test drug on cerebral blood flow. PET measurements should be performed at physiological steady state, i.e., not when cerebral blood flow undergoes (rapid) changes due to the test drug.

**Fig. 2:**
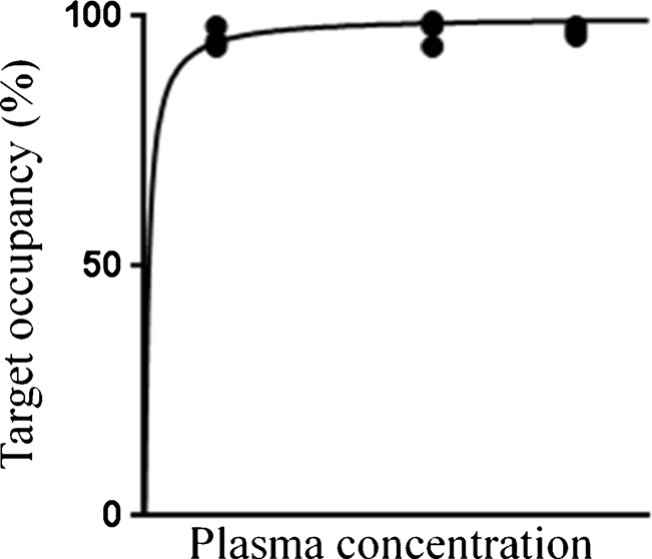
Example of occupancy results following administration of three different doses of the test drug. In this particular case, drug doses were fixed prior to the start of the PET study and almost all occupancy values were close to 100 %, providing little information on the upslope of the binding curve. This can be avoided by the use of an adaptive design, where each drug dose is based on the results of the previous measurement(s)

In some cases, e.g., because of safety issues, only a limited range of doses of the test drug can be investigated in a human occupancy study, precluding a full assessment of the relationship between target occupancy and drug dose. If occupancies are obtained only at high (i.e. >95 %) or low (i.e. <10 %) levels, it might be difficult to assess whether this is due to technical problems in the quantification of the occupancy or due to the non-optimal dose selection of the test drug. If preclinical PET occupancy data are available for a wider range of occupancies, the missing human occupancy data may be extrapolated, provided the animal data can be extrapolated to the human situation. In clinical PET occupancy studies, especially when preclinical PET occupancy data are not available, the doses of the test drugs should ideally be determined using an adaptive design. Figure 2 shows an example of a rather poorly defined dose-occupancy curve, which was obtained using doses of the test drug that had been fixed before the start of the PET study. Such an ill-defined dose-occupancy curve can be avoided by using an adaptive design.

### PET data collection

As for quantitative brain PET studies, dynamic PET measurements are required for an occupancy study. Depending on the selected (required) quantitative analytical method for the radioligand, arterial blood sampling, metabolite analysis for the parent fraction of radioligand and measurement of protein binding may be needed. After administration of the drug under investigation, changes in brain kinetics of the radioligand may occur due to peripheral blocking and/or changes in metabolite profile and protein binding. When a reference tissue model can be used, it is important to assess whether the proposed reference tissue can still be used as reference tissue after administration of the drug. In case of the pretreatment PET scan, one or more venous blood samples are taken to measure the plasma concentration of the test drug. As the plasma concentration of the test drug is expected not to change too much during a PET measurement, often the plasma concentration just before the start of the PET measurement is used as the actual plasma drug concentration. However, blood samples at the end of the PET scan should also be collected to make sure that the plasma drug concentration has not changed too much and, in some cases, the mean of the plasma concentrations at start and end of a PET scan are used for the pretreatment plasma drug concentration.

### PET data analysis

#### Calculation of outcome measures for radioligand binding

Calculation of outcome measures for PET occupancy studies is based on established quantitative methods for individual radioligands. For a full description of the nomenclature of the outcome measures used for quantification, see Innis et al. [4].

A general procedure of PET data analysis is as follows. First, for each individual subject, an MRI scan is acquired. Next, a summed PET image is generated from the dynamic PET scan to enable co-registration of PET and MRI images. After co-registration, the MRI is used to delineate regions of interest (ROIs). Projecting these ROIs on the (co-registered) PET scan then generates corresponding time-activity curves.

If studies are performed using a radioligand for which a region in the brain exists that is devoid of the target being studied (a so called reference region), reference tissue models can be used such as the simplified reference tissue model (SRTM), the Logan non-invasive method, or various multilinear reference tissue models, depending upon which method has been validated for the quantification of the radioligand being used [9–11].

When a radioligand is used for which no reference region exists, the only option is to perform kinetic analysis using a metabolite corrected arterial input function. In general, only V_T_ can be obtained as outcome measure (i.e., including a non-specific component), as direct estimates of *BP*_ND_ and *V*_S_ tend to be less robust (i.e., with large standard errors).

Although in some cases SUV or SUVr have been used as surrogate outcome measures within the context of an occupancy study [12], in general this approach is questionable, as SUV and to some extent SUVr depend on delivery (plasma curve, perfusion), which may be affected by the drug. As for any simplification, this approach can only be used after full validation against full kinetic analysis.

Automated ROI delineation with or without partial volume correction can be used for analysing PET occupancy data. Parametric occupancy images can be generated as well. However, ROI delineation and partial volume correction should be exactly the same for both baseline and pretreatment PET scans.

#### Calculation of the target occupancy

As mentioned above, in case of radioligands for which a reference region exists, *BP*_ND_ is the outcome measure. Using *BP*_ND_, occupancy Occ can be calculated as follows:

1 $$ \mathrm{O}\mathrm{c}\mathrm{c}\left(\%\right)=100\times \left(B{P_{\mathrm{ND}}}^{\mathrm{baseline}}{\textstyle \hbox{-} }B{P_{\mathrm{ND}}}^{\mathrm{drug}}\right)/B{P_{\mathrm{ND}}}^{\mathrm{baseline}}, $$

where *BP*_ND_^baseline^ is *BP*_ND_ under baseline conditions and *BP*_ND_^drug^ that after administration of the test drug.

In case of radioligands for which no reference region exists, *V*_T_ is calculated as outcome measure. However, *V*_T_ consists of a specifically bound part (*V*_S_) and a nondisplacable part (*V*_ND_), which means that occupancy is not directly proportional to the change in *V*_T_. There are several methods to estimate *V*_ND_, for example, the method proposed by Lassen et al. [13] in which *V*_ND_ is derived from the various *V*_T_ values obtained in a PET occupancy study. Although this method is easy to apply, it is important to check whether the underlying assumptions of identical occupancy levels and *V*_ND_ values across the brain are still valid for the study.

For radioligands with irreversible binding to the target, such as [^11^C]-L-deprenyl-D_2_ (monoamine oxidase type B (MAO-B), k_3_ or λk_3_ can be used as outcome measure to be used in Eq. 1 for calculating inhibition (λ = K_1_/k_2_) [14, 15].

#### Estimation of K_d_^plasma^ of the test drug

After occupancy values have been calculated from the PET measurements, they can be plotted against administered dose and/or plasma level of the test drug. Based on the Hill equation [16], the plotted data can be fitted to the following equation,

2 $$ \mathrm{O}\mathrm{c}\mathrm{c}\ \left(\%\right) = {{\mathrm{C}}_{\mathrm{P}}}^{\mathrm{d}\mathrm{rug}}/\ \left({{\mathrm{C}}_{\mathrm{P}}}^{\mathrm{d}\mathrm{rug}}+{{\mathrm{K}}_{\mathrm{d}}}^{\mathrm{plasma}}\right) \times \mathrm{O}\mathrm{c}{\mathrm{c}}_{\mathrm{max,}} $$

where Occ_max_ represents the maximum occupancy and C_P_^drug^ is the plasma concentration of the test drug or, alternatively, the dose administered.

Occ_max_ might be fixed to 100 % or estimated based on the actual data. K_d_^plasma^ corresponds to the plasma concentration (or dose) at which the target occupancy is 50 %. The equation was originally based on K_d_, which is the dissociation constant for the local concentration at the target binding. In practice, K_d_^plasma^ is used as a surrogate of K_d_ based, assuming that the two are correlated.

As it is not guaranteed that the relationship between radioligand and test drug can be described by a Hill equation, pharmacological information on both radioligand and test drug should be considered before applying Eq. 2.

### PET occupancy studies as a dose selection tool for clinical trials

**Fig. 3:**
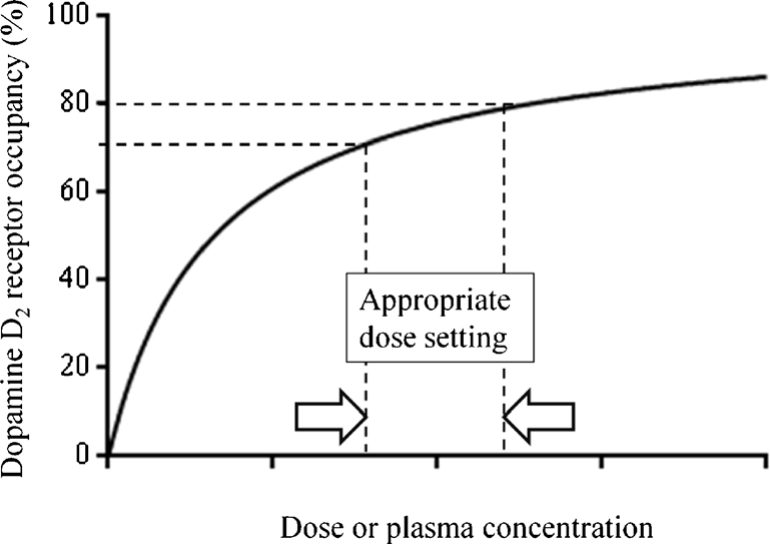
Schematic demonstration of the relationship between the target occupancy and the dose or plasma concentration of a drug using dopamine D_2_ receptor and antipsychotics as example. In case of dopamine D_2_ receptor, more than 70 % occupancy is necessary for clinical efficacy, but extrapyramidal symptoms emerge at more than 80 % occupancy. The dose or plasma concentration to occupy 70–80 % of the dopamine D_2_ receptor is considered to be appropriate for the drug treatment

**Fig. 4:**
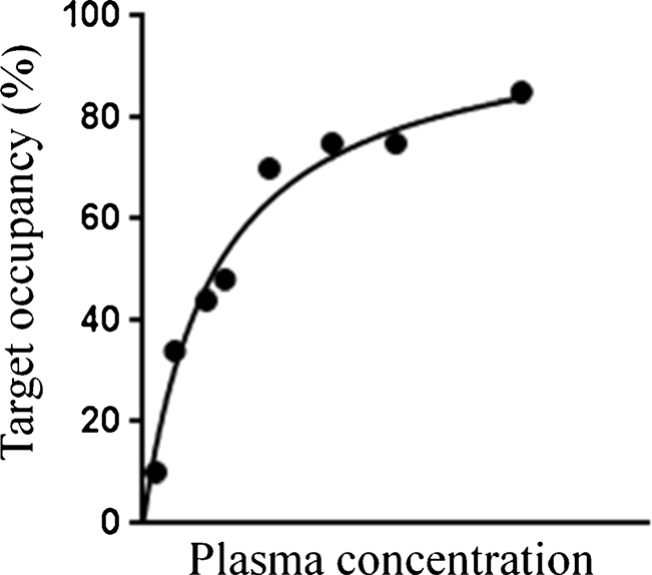
Example of eight occupancy data points plotted against the plasma concentration of the test drug. If the doses of the test drug are selected appropriately (adaptive design), a wide range of occupancies can be covered, which would help to select the doses for further clinical trials

**Fig. 5:**
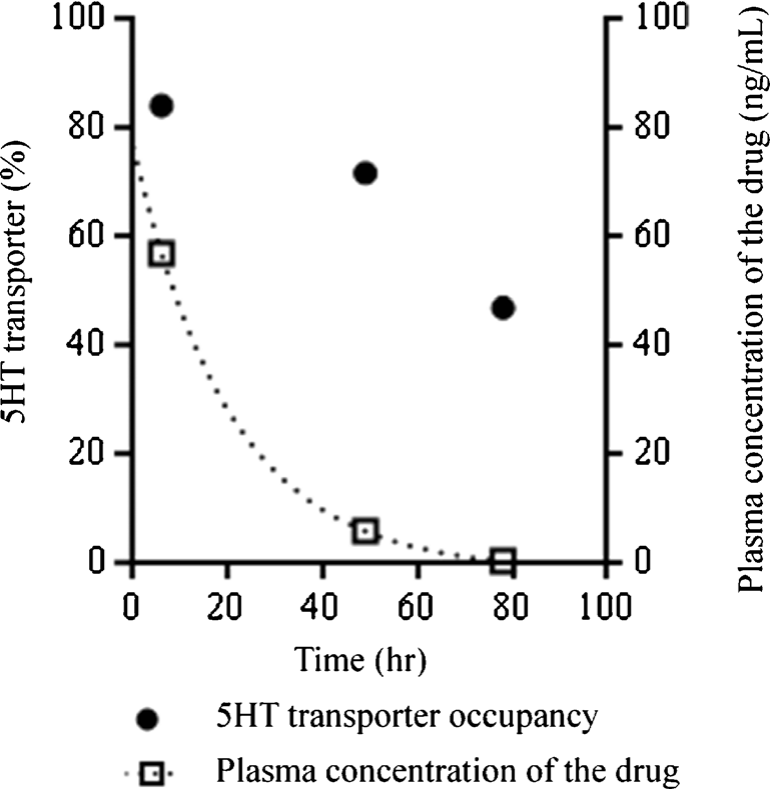
Schematic demonstration of the time course of the target occupancy and the plasma concentration of a drug using 5HT transport and antidepressant. The time course of the target occupancy can help to decide the dosing regimen in addition to the time course of the plasma concentration of the drug

The relationship between target occupancy by a drug and its clinical efficacy and/or presence of side effects has been established successfully for a limited number of targets, such as the dopamine D_2_ receptor and the 5-HT transporter [17–19]. For example, in case of the dopamine D_2_ receptor, approximately 70 % occupancy is necessary for clinical efficacy, but more than 80 % occupancy is related to the extrapyramidal symptoms (Fig. 3) [17]. For new drug targets, such a relationship should ideally be established in phases 2 and 3 of the drug development program. Based on the relationship between test drug’s dose and the target occupancy, as shown in Fig. 4, several doses of a test drug can be chosen for further clinical trials, e.g. ,the first dose could correspond with 20 % target occupancy, the second with 50 % and the third with 80 %. If the appropriate dose to show the clinical efficacy is not known beforehand, this procedure will provide the most efficient approach to obtain maximal clinical efficacy together with minimal side effects. In other words, a PET occupancy study is an ideal way to select an appropriate drug dose for further clinical trials. In any case, it provides a means to avoid selecting too high or too low doses. Finally, by performing PET measurements at various times after treatment, the time course of target occupancy can be established, which helps in the selection of the optimal dosing regimen of the test drug (Fig. 5) [20, 21].

Finally, as drug development in pharmaceutical companies is usually performed under tight time constraints, appropriate PET occupancy study protocols based on these guidelines are expected to help to speed up the drug development.
